# Supplementary material for: An expanded reference map of the human gut microbiome reveals hundreds of previously unknown species
Source: Nat Commun. 2022 Jul 5;13:3863. doi: 10.1038/s41467-022-31502-1 (PMC9256738; doi:10.1038/s41467-022-31502-1)
Supplement: Supplementary file 1 — Supplementary Information [file 41467_2022_31502_MOESM1_ESM.pdf]

Supplementary Information:

An Expanded Reference Map of the Human Gut Microbiome Reveals Hundreds of Previously Unknown Species

S. Leviatan et al.

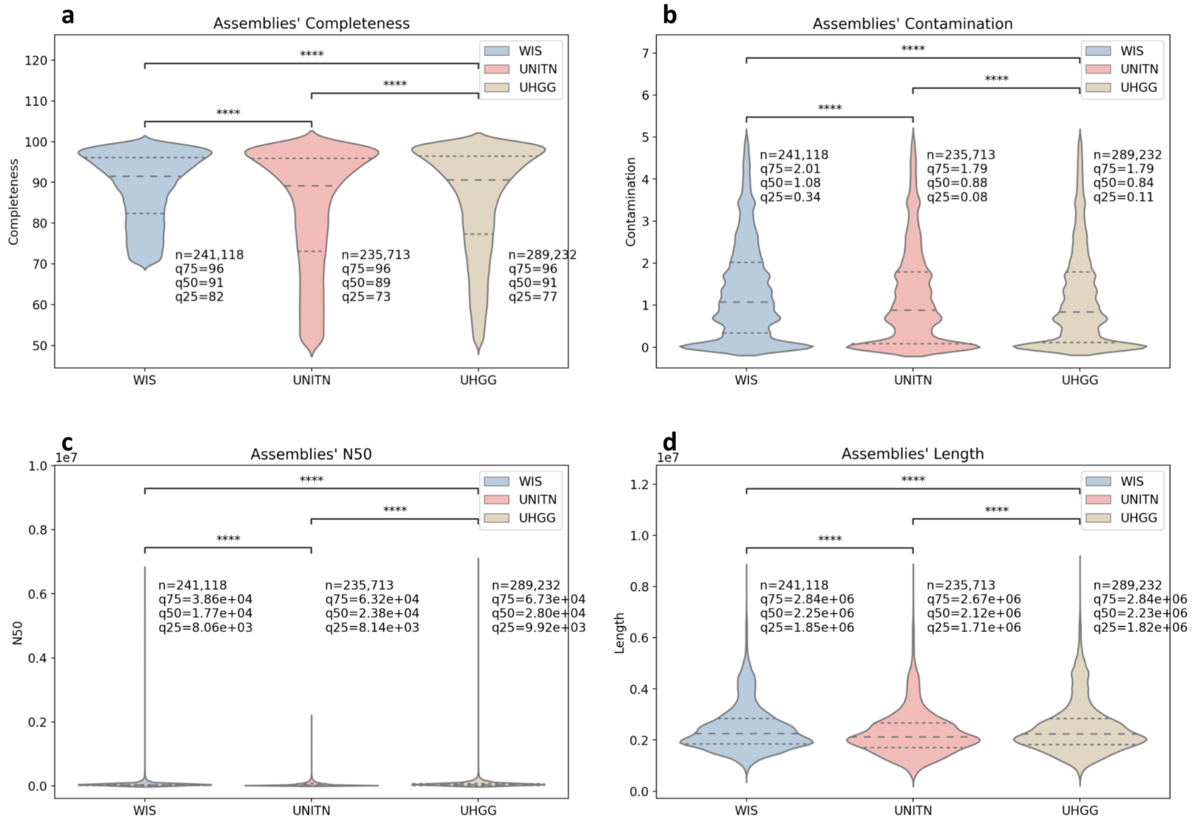

Supplementary Figure 1. The set of assemblies used by WIS shows higher quality measures than the set used by UNITN

WIS (n=241,118 genomes) in blue, UNITN (n=235,713) in red and UHGG (n=289,232) in brown. **a-d** are violin plots of the completeness, contamination, N50 and length of the genomes, respectively. In each plot, the y-axis is the parameter value and drawing width is the kernel density estimate. The dashed line is the median and the dotted lines are the interquartile range. Bonferroni corrected p-value annotations: not significant (ns) q>0.05, *q<0.05, **q<0.01, ***q<0.001, ****q<0.0001 according to Mann-Whitney U test. Completeness threshold in WIS was 70% while it was 50% in UNITN and UHGG. Panels b and c seemingly have values under zero, this is a graphical illusion of violin plots since the distribution is dense around the minimal value (zero).

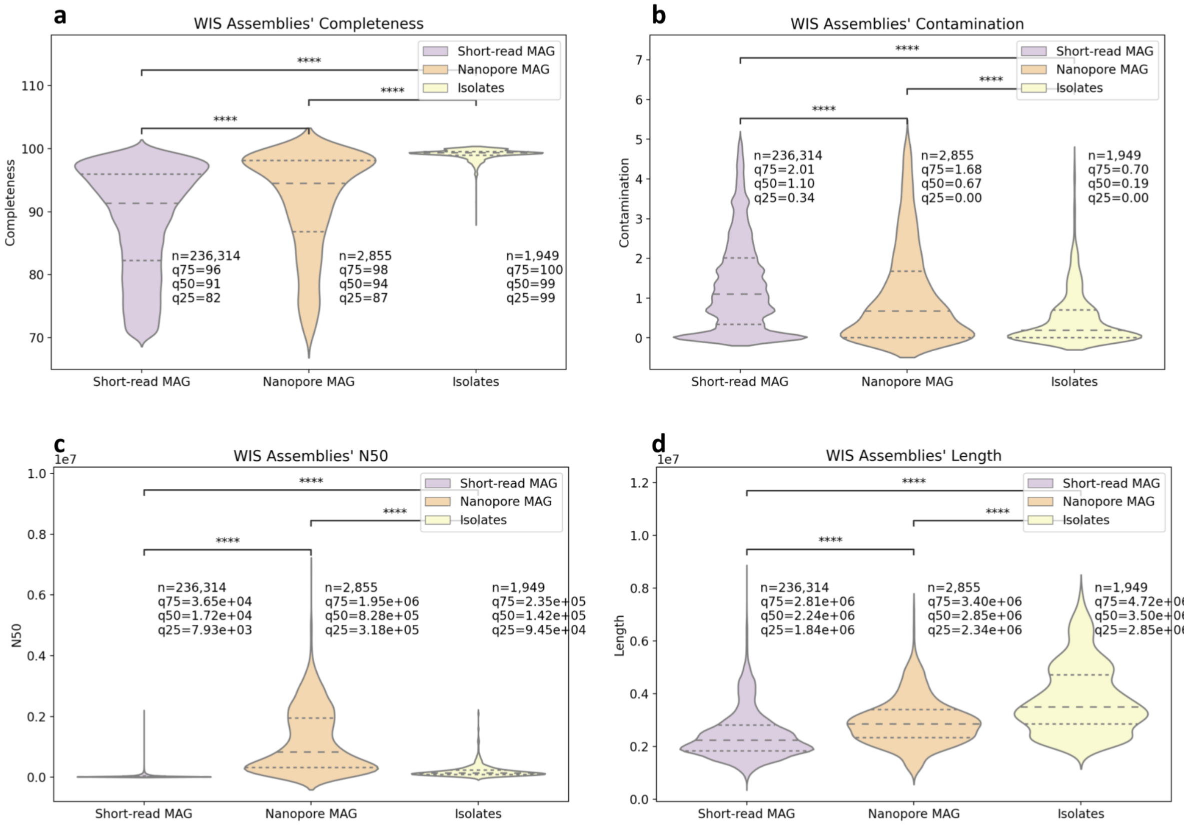

Supplementary Figure 2. Nanopore and isolates assembled genomes are improved in all quality measures considered to the short-read based MAG

Short-read metagenome-assembled genome (MAG) (n=236,314 genomes) in purple, nanopore MAG (n=2,855) in orange and isolates (n=1,949) in yellow. **a-d** are violin plots of the completeness, contamination, N50 and length of the genomes, respectively. In each plot, the y-axis is the parameter value and drawing width is the kernel density estimate. The dashed line is the median and the dotted lines are the interquartile range. Bonferroni corrected p-value annotations: not significant (ns) q>0.05, *q<0.05, **q<0.01, ***q<0.001, ****q<0.0001 according to Mann-Whitney U test. Panels b and c seemingly have values under zero, this is a graphical illusion of violin plots since the distribution is dense around the minimal value (zero).

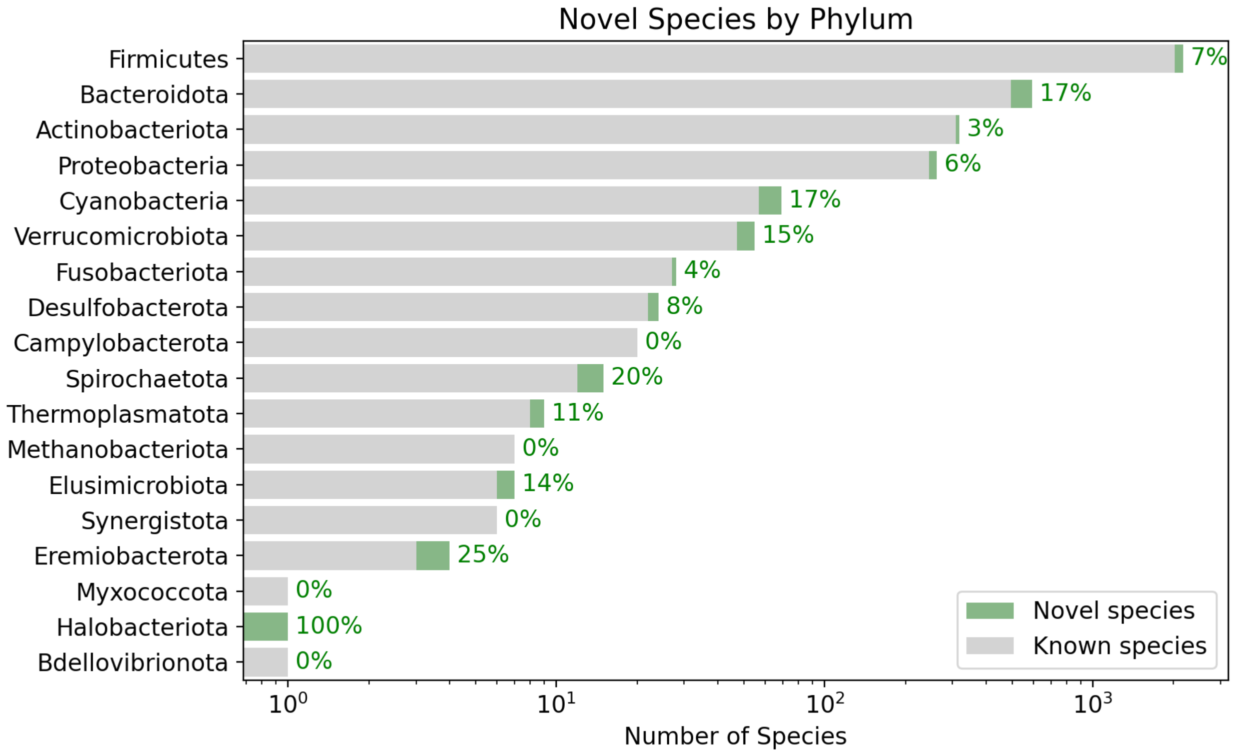

Supplementary Figure 3. Novel species by phylum

Number of known and novel species in each phylum. Percentage to the right of the bar is the proportion of novel species out of all the species of the phylum (within our reference set).
