# Supplementary material for: An expanded reference map of the human gut microbiome reveals hundreds of previously unknown species
Source: Nat Commun. 2022 Jul 5;13:3863. doi: 10.1038/s41467-022-31502-1 (PMC9256738; doi:10.1038/s41467-022-31502-1)
Supplement: Supplementary file 3 — Description of Additional Supplementary Files [file 41467_2022_31502_MOESM3_ESM.pdf]

File Name: Supplementary Data 1
Description: Metadata of all 241,118 assemblies used in clustering.
Columns of table are:
species - species level cluster ID
genus - genus level cluster ID
family - family level cluster ID
Completeness - based on checkm
Contamination - based on checkm
#contigs - number of contigs, based on checkm
N50 - based on checkm
Length - assembly length in bp, based on checkm
#assemblies - only for representatives, the number of assemblies in the species level cluster
Clade Separation Score - only for representatives, based on GUNC
Reference Representation Score - only for representatives, based on GUNC
representative - True if this assembly is the representative of the species level cluster
source - data source Segal/Segata/Kyrpides/Xiao/Lawley/Alm
type - type Short-read MAG/Nanopore MAG/Isolate
d,p,c,o,f,g,s - only for representatives, taxonomy based on GTDB-tk classification
avg_UNITN_sgb - only for representatives, closest UNITN SGB, by average distance, based of phylophlan
avg_UNITN_dist - only for representatives, distance to avg_UNITN_sgb, based of phylophlan
min_UNITN_sgb - only for representatives, closest UNITN SGB, by minimal MASH distance to representatives
min_UNITN_dist - only for representatives, MASH distance to min_UNITN_sgb
min_UHGG_sgb - only for representatives, closest UHGG SGB, by minimal MASH distance to representatives
min_UHGG_dist - only for representatives, MASH distance to min_UHGG_sgb
ParticipantID - unique ID of the participant from which the sample was taken, and the assembly was built
SampleID - unique ID of the sample the assembly was built from
AssemblyID - unique ID of the assembly fasta file

File Name: Supplementary Data 2
Description: Validation sets mapping percentage.
Columns of table are:
% Read Mapping WIS - percentage of reads aligned to WIS reference set
% Unique Read Mapping WIS - percentage of reads best aligned to a single position in the WIS reference set
% Read Mapping UNITN - percentage of reads aligned to UNITN reference set
% Unique Read Mapping UNITN - percentage of reads best aligned to a single position in the UNITN reference set
% Read Mapping EMBL - percentage of reads aligned to EMBL reference set
% Unique Read Mapping EMBL - percentage of reads best aligned to a single position in the EMBL reference set

validation - country of validation sample, from Israel 3,096 samples, Netherlands 1,528, India 110, El Salvador 113 or Tanzania 68

File Name: Supplementary Data 3
Description: Genetic annotations of WIS representatives.
Due to the size of the file it is only available via figshare
Columns of table are:
GeneID - unique ID of each gene
Rep - representative genome species ID
contig, start_pos, end_pos, strand, feature, gene, product - prokka produced information about the gene
ID - prokka and eggnog shared ID
The rest of the columns - eggnog produced information about the gene

File Name: Supplementary Data 4
Description: Validation sets species' relative abundances.
Each row is a validation sample (Israel 3,096 samples, Netherlands 1,528 samples) and each column a species ID. Each cell is the relative abundance estimation for the pair of species and sample, or empty if it does not exist (over a 10^{-4} detection threshold). Per sample all relative abundances sum to 1.

File Name: Supplementary Data 5
Description: Antibiotic resistances of WIS representatives.
Columns of table are:
sequence, start, end, strand, gene coverage, coverage_map, gaps, perc_coverage, perc_identity, database, accession, product, resistance_database - ABRicate produced information
resistance_comb - combined resistance_database columns separated by "|"
resistance_final - drug resistances at most specific level annotated separated by ";"
drug_class - higher level drug classes (based on resistance_final)
broad_drug_class - all beta-lactam drug classes consolidated to one group
The rest of the columns - 1 or 0 for presence or absence of the term in the above 3 categories
