# Supplementary material for: An expanded reference map of the human gut microbiome reveals hundreds of previously unknown species
Source: Nat Commun. 2022 Jul 5;13:3863. doi: 10.1038/s41467-022-31502-1 (PMC9256738; doi:10.1038/s41467-022-31502-1)
Supplement: Supplementary file 10 — Reporting Summary [file 41467_2022_31502_MOESM10_ESM.pdf]

nature portfolio

Corresponding author(s): Sigal Leviatan

Last updated by author(s): Jun 14, 2022

Reporting Summary

Nature Portfolio wishes to improve the reproducibility of the work that we publish. This form provides structure for consistency and transparency in reporting. For further information on Nature Portfolio policies, see our Editorial Policies and the Editorial Policy Checklist.

Statistics

For all statistical analyses, confirm that the following items are present in the figure legend, table legend, main text, or Methods section.

n/a	Confirmed	
☐	☒	The exact sample size (n) for each experimental group/condition, given as a discrete number and unit of measurement
☐	☒	A statement on whether measurements were taken from distinct samples or whether the same sample was measured repeatedly
☐	☒	The statistical test(s) used AND whether they are one- or two-sided<br>*Only common tests should be described solely by name; describe more complex techniques in the Methods section.*
☒	☐	A description of all covariates tested
☐	☒	A description of any assumptions or corrections, such as tests of normality and adjustment for multiple comparisons
☐	☒	A full description of the statistical parameters including central tendency (e.g. means) or other basic estimates (e.g. regression coefficient) AND variation (e.g. standard deviation) or associated estimates of uncertainty (e.g. confidence intervals)
☐	☒	For null hypothesis testing, the test statistic (e.g. F, t, r) with confidence intervals, effect sizes, degrees of freedom and P value noted<br>*Give P values as exact values whenever suitable.*
☒	☐	For Bayesian analysis, information on the choice of priors and Markov chain Monte Carlo settings
☒	☐	For hierarchical and complex designs, identification of the appropriate level for tests and full reporting of outcomes
☒	☐	Estimates of effect sizes (e.g. Cohen's d, Pearson's r), indicating how they were calculated

Our web collection on statistics for biologists contains articles on many of the points above.

Software and code

Policy information about availability of computer code

Data collection	SPAdes (version 3.10.1) with argument '--only-error-correction' was run for preliminary error correction.<br>Megahit (version 1.1.1) with argument '--min-contig-len 1000' was run to build contigs.<br>Bowtie2 (version 2.2.9) was run to build an index from the contigs file with default arguments, and to map the original sample to this index, with '--very-sensitive-local' argument.<br>SAMTools (version 1.3.1) was used to create a sorted bam from the bowtie output.<br>Metabat2 (version 2.12.1) with argument '-m 1500' was run, with a depth file created using jgi_summarize_bam_contig_depths with default arguments, to bin contigs into assemblies.<br>CheckM lineage_wf (version 1.0.13) with default arguments was used to determine quality parameters.<br>Prokka (version 1.12) with default arguments was used to create gff files for each assembly which passes initial quality thresholds.<br>CMSeq (version 1.2) polymut with arguments '--mincov 10 --minqual 30 --dominant_frq_thrsh 0.8' was used to assess heterogeneity.
Data analysis	All of the source code used to create our reference set is available in the following git repository:<br>https://github.com/erans99/GutReferenceSet<br><br>all code use python libraries: pandas 1.2.5, numpy 1.21.0, matplotlib 3.4.3, fastcluster 1.2.3, scipy 1.7.0, seaborn 0.11.1<br>and the tools:<br>CheckM lineage_wf (version 1.0.13)<br>prodigalv2.6.3<br>hmmer version bin-3.2<br>pplacer-Linux-v1.1.alpha19<br>mash-Linux64-v2.1<br>bowtie2 version 2.3.4.2

prokka version 1.14.6
eggnog version 2.0.4
GTDB-tk version 1.7.0, database version R202
phylophlan_metagenomic version 3.0.35
Genome UNClutterer (GUNCr) version 1.0.5
ABRicate tool version 1.0.1

For manuscripts utilizing custom algorithms or software that are central to the research but not yet described in published literature, software must be made available to editors and reviewers. We strongly encourage code deposition in a community repository (e.g. GitHub). See the Nature Portfolio guidelines for submitting code & software for further information.

Data

Policy information about availability of data

All manuscripts must include a data availability statement. This statement should provide the following information, where applicable:
- Accession codes, unique identifiers, or web links for publicly available datasets
- A description of any restrictions on data availability
- For clinical datasets or third party data, please ensure that the statement adheres to our policy

A single fasta file of all the 3,594 species representatives genomes, and all supplementary files can be downloaded from:
https://doi.org/10.6084/m9.figshare.16885261

The 142,912 assembled genome fastas, divided into 10 tarred and gzipped files, and the full metadata file describing these assemblies is available at:
https://doi.org/10.5281/zenodo.5767857

Field-specific reporting

Please select the one below that is the best fit for your research. If you are not sure, read the appropriate sections before making your selection.

☒ Life sciences ☐ Behavioural & social sciences ☐ Ecological, evolutionary & environmental sciences

For a reference copy of the document with all sections, see nature.com/documents/nr-reporting-summary-flat.pdf

Life sciences study design

All studies must disclose on these points even when the disclosure is negative.

Sample size	No statistical method was used to predetermine sample size, this is a meta-analysis were we took all available data (up to 2019) after quality control and biases reduction as described above.
Data exclusions	Genome assemblies had to be over 70% complete, under 5% contaminated. No two assemblies of the same person and species were used, the one with the highest quality was included. Only gut assemblies were used. If not metadata was available to determine these criteria assembly was excluded.
Replication	n/a, as this is not an experiment. Any new assemblies produced, by us or others, should be added to this set to create a richer set of assemblies and cluster together, thus refining our knowledge of the species clusters. And not instead of used data.
Randomization	n/a, as this is not an experiment there is no allocation to randomize.
Blinding	n/a, as this is not an experiment there is no allocation to hide.

Reporting for specific materials, systems and methods

We require information from authors about some types of materials, experimental systems and methods used in many studies. Here, indicate whether each material, system or method listed is relevant to your study. If you are not sure if a list item applies to your research, read the appropriate section before selecting a response.

Materials & experimental systems

n/a	Involved in the study	
☒	☐	Antibodies
☒	☐	Eukaryotic cell lines
☒	☐	Palaeontology and archaeology
☒	☐	Animals and other organisms
☐	☒	Human research participants
☒	☐	Clinical data
☒	☐	Dual use research of concern

Methods

n/a	Involved in the study	
☒	☐	ChIP-seq
☒	☐	Flow cytometry
☒	☐	MRI-based neuroimaging

Human research participants

Policy information about studies involving human research participants

Population characteristics	Israel (90%) and USA (10%), Adults (99%) and children (<1%), Female (61%) and male (39%), mostly of healthy individuals.
Recruitment	No recruitment was done for this study as this is a meta-anlysis. Study uses the sequencing data of metagenomic samples created by prior studies.
Ethics oversight	The study was approved by the Institutional Review Board (IRB) ethics committee of the Weizmann Institute of Science (Reference number: 1719-1).

Note that full information on the approval of the study protocol must also be provided in the manuscript.
